# An Inverse-Linear Logistic Model of The Main Sequence

**DOI:** 10.16910/jemr.10.3.4

**Published:** 2017-05-29

**Authors:** Andrew T. Duchowski, Krzysztof Krejtz, Cezary Biele, Anna Niedzielska, Peter Kiefer, Ioannis Giannopoulos, Nina Gehrer, Michael Schönenberg

**Affiliations:** Clemson University Clemson, SC, USA; SWPS University of Social Sciences & Humanities Warsaw, Poland; National Information Processing Institute Warsaw, Poland; ETH Zürich Zürich, Switzerland; Universität Tübingen Tübingen, Germany

**Keywords:** saccades, microsaccades, main sequence, non-linear modeling

## Abstract

A model of the main sequence is proposed based on the logistic function. The model’s fit to the peak velocity-amplitude relation resembles an S curve, simultaneously allowing control of the curve’s asymptotes at very small and very large amplitudes, as well as its slope over the mid-amplitude range. The proposed inverse-linear logistic model is also able to express the linear relation of duration and amplitude. We demonstrate the utility and robustness of the model when fit to aggregate data at the smalland mid-amplitude ranges, namely when fitting microsaccades, saccades, and superposition of both. We are confident the model will suitably extend to the largeamplitude range of eye movements.

## Introduction

Several models characterizing the relationship between saccadic peak velocity and amplitude have been proposed, including the power law, an exponential curve, and an inverse-linear model. Each of these can be made to express the interdependence of the main sequence parameters of amplitude, duration, and peak velocity. However, no model appears to adequately span a wide range of amplitudes. Some models perform better at the small-amplitude range others do better at the mid-amplitude range while others are better suited to the large-amplitude range.

To derive a robust model of the main sequence, we propose fitting the saccadic peak velocity-amplitude relation with an S curve such that peak velocity displays a fairly flat slope over very small and very large amplitudes. Meanwhile, the relation of saccadic peak velocity to duration and amplitude suggests that the model should *also* conform to the linearity of the duration-amplitude relation. Finally, parameters of the model should be easy to interpret. In this paper, we derive the S curve model from the logistic function and show how this model satisfies all of the aforementioned requirements. To satisfy the criterion of interdependence between non-linear peak velocity and linear duration relations to amplitude, the model requires an inverse-linear component, producing an inverse-linear logistic model, suitable for expressing both relations.

We demonstrate the utility and robustness of the model when fit to *aggregate* data collected from three experiments at three different laboratories, utilizing two different eye trackers. The first two experiments required maintenance of steady gaze while the third did not. Using saccade and microsaccade detection algorithms based on the work of Engbert and colleagues (13, 11, 12 ), we show how our model provides superior fits to peak velocity amplitude relations of microsaccades, saccades, and to the superposition of both. Our model is simultaneously capable of providing a linear fit to duration-amplitude indistinguishable from a fit provided by the established linear main sequence (for an example of a linear fit to aggregate microsaccade data, see Siegenthaler et al. (29).

Our work is similar to that of Diaz-Piedra et al. (10), who also model the saccade peak velocity amplitude relation nonlinearly and test different fits to individuals’ data (using a first-order polynomial and power-law fits). We model and empirically test our inverse-linear logistic S curve fit against three other fits (power-law, exponential, and inverse-linear) and find that ours provides the best statistical fit. Unlike DiazPiedra et al., we fit our data to the aggregate collection microsaccade and saccade data from many participants instead of fitting the function per individual. Diaz Piedra et al. also do not appear to consider the reciprocal of their velocity-amplitude fits, i.e., they do not show whether the nonlinear fits they use for velocityamplitude can also simultaneously be used to model the *linear* duration-amplitude relation. Due to the interdependence of main sequence parameters pointed out by Lebedev, Van Gelder, and Tsui (19), any non linear function chosen to model the velocity-amplitude relation should *also* be suitable as a model of the linear duration-amplitude relation. Below we first go through the mathematical derivation of our inverse-linear logistic model, showing how it can serve to model both relations, then we describe the three experiments whose data we test our four function fits on.

## Saccadic Characteristics

**Figure 1:**
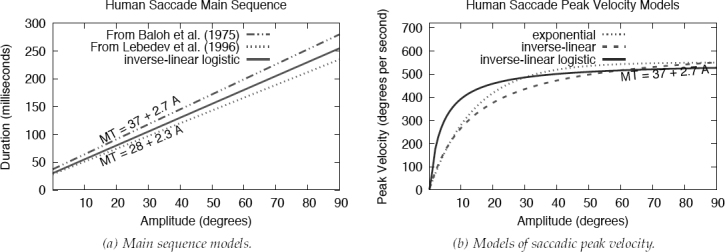
Interdependence of saccadic main sequence parameters: (a) linear duration-amplitude expressions from Baloh, Sills, Kumley, and Honrubia (3), Collewijn, Erkelens, and Steinman (8), and our inverse-linear logistic function (see text); (b) relation of saccadic peak velocity to amplitude matching duration-amplitude relation in (a), modeled as exponential expression provided by Baloh et al. (3), an inverse-linear derivation, and our inverse linear logistic expression (see text).

Human saccades are stereotyped (15) and presumed to be ballistic (6), meaning programmed motor movements whose trajectory is unchangeable once in flight. Saccades follow the *main sequence* describing the relationship between saccadic peak velocity and amplitude (Bahill, Clark, & Stark, 2; Baloh et al., 3; Knox, 18). The main sequence *also* relates saccade duration to amplitude. Treating saccade duration as movement time MT, the main sequence can be expressed as a linear relation


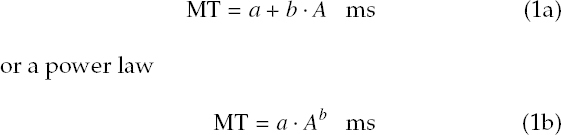


where amplitude A is given in degrees visual angle in both instances (Becker, 4). Example fits are given for the power law (1b) by Yarbus (34) with a = 0.021 and b = 0.4 and for the linear relation (1a) by Baloh et al. (3) with a = 37 and b = 2.7, in milliseconds and milliseconds/degree, respectively, see Figure 1(a)^1^ The lower limit (a) of saccade duration is due to finite risetime of muscle fibre twitches (for microsaccades of amplitude less than 0.5°, duration of 14 ms have been reported (Becker, 4)). The relationship between duration and saccade amplitude is normally linear for saccades of up to about 80° (although most naturally occurring saccades range up to about 15°-20° (Bahill et al., 2) and up to 30° without head movement (Lebedev et al., 19)).

Unlike the linear relation of duration-amplitude, the peak velocity of saccadic eye movements is related in a nonlinear manner to their amplitude over a thousand fold range (from 3’ minutes of arc to 50° ), with data scatter noted as “extremely small” (Bahill et al., 2). Peak velocity is related in a quasi-linear manner to saccadic amplitude up to about 15° or 20°, when a soft saturation limit is reached. Lebedev et al. (19) provide an inverse-linear model (the Michaelis-Menten equation) where peak velocity V_p_ is modeled by


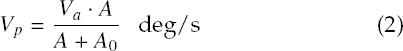


where A denotes saccade amplitude, V_a_ is an asymptotic maximum of peak velocity (the saturation value, in degrees per second), and A*0* is the half-maximum amplitude (in degrees), i.e., the amplitude at which 50% of the peak velocity is reached. Baloh et al. (3) compare alternative peak velocity models using a power-law equation


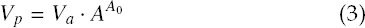


and an exponential curve


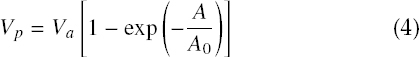


were *A*_0_ in this latter form represents the amplitude for which peak velocity reaches 63% of its saturation (Lebedev et al., 19), see Figure 1(b) where the exponential model is plotted for *v*_a_ = 551 degrees/second and *A*_0_ = 14 degrees as fit by Baloh et al. (3) to their observations, corresponding to their MT = 37 + 2.7 A duration-amplitude regression shown in Figure 1(a).

The interdependence between the dynamic properties of saccadic amplitude, duration, and peak velocity might not be immediately obvious. By itself, the main sequence does not provide a complete description of the saccadic system, which as a whole, is non-linear (Van Opstal & Van Gisbergen, 31). Saccades of different amplitudes have differently shaped velocity profiles. The velocity profile of small saccades is symmetrical while it is skewed for large saccades, and can be modeled by the expression


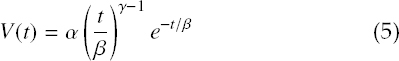


where time t≥0, α, β>0 are scaling constants for velocity and duration, respectively, and $$$ is the shape parameter that determines the degree of asymmetry. Small values of *γ* yield asymmetrical velocity profiles and as *γ* tends to infinity, the function assumes a symmetrical (Gaussian) shape see Van Opstal and Van Gisbergen (31) as well as Baloh et al. (3) or Collewijn et al. (8) for illustrations.

Interdependence of the main saccadic parameters of amplitude, duration, and peak velocity is explained by a strong linear relationship (r > 0.98) between mean velocity V_m_ and peak velocity *V_p_* (Lebedev et al., 19). By definition, the mean velocity of saccades is computed directly from their duration and amplitude,


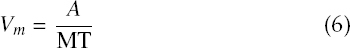


where solving for 
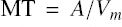
 and equating with (1a) leads to the inverse-linear dependence of mean velocity on amplitude,


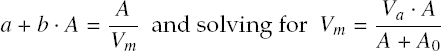


produces the Michaelis-Menten equation (2) for Vm with 
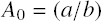
 and 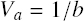 (Becker, 4). Similarly, equating (6) with (1b)

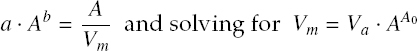



leads to the power-law equation (3) for Vm with A_0=(1-b) and_ V_a=(1/a)_

Peak velocity initially rises in proportion to saccade amplitude and then saturates as the amplitude becomes larger. Plotted as a function of saccade amplitude, V_p_ resembles a scaled-up version of V_m_,

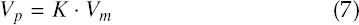



where K is constant (Becker, 4; Lebedev et al., 19).

As a numerical example, solving the main sequence provided by Baloh et al. (3) MT = 37+2.7A=A/V_m_ for V_m_ produces the Michaelis-Menten inverse-linear dependence of peak velocity on amplitude (2) with A_0_ = 13.70 and V_a_ = 0.37 · 551 where 0.37 is 1/b from (1a) and 551 is the asymptotic maximum peak velocity used in (4). Using (7), we found a good approximation to (4) by setting K = 3.11, see Figure 1(b). For a numerical example of the power law, see Lebedev et al. (19).

In their comparison of various models, including the inverse-linear, exponential, and power-law models of saccadic peak velocity, Lebedev et al. (19) note that the inverse-linear and exponential models are of no use outside the range of estimation (i.e., < 1.5° or > 30°) and that their parameters do not allow any reasonable interpretation. They give an approximation of (horizontal) saccadic eye movements’ peak velocity in the form of a square-root model, effectively Yarbus’ power-law model given in (6) with A*o* fixed to 1/2. They claim that the square-root model is adequate for a range of amplitudes from 1.5° to 30°, i.e., the mid-amplitude range. Furthermore, they show that all models except the square-root model are unstable with respect to a small shift of the amplitude ranges from which they were drawn. Meanwhile, within the 7°-15° range of amplitudes, they found the power-law model served better than the square-root model with respect to goodness of fit, with the inverse-linear and exponential models unacceptably unstable.

From the model comparison of Lebedev et al. (19), it appears no model provides a good fit to the data across a wide range of saccadic amplitudes. They note that the interdependence of the main sequence parameters, specifically relations (6) and (7), allows categorization of saccades into three ranges of saccade amplitudes: the small-amplitude range (A < 1.5°) in which duration remains fairly constant and increase in amplitude is caused by an increase in peak velocity; the midamplitude range (1.5° ≤*A* ≤ 35°) in which the increase in the amplitude is caused by an increase in saccade duration and peak velocity; and the large-amplitude range (35^0^A) in which peak velocity saturates such that an increase in amplitude is caused primarily by duration.^2^ The majority of naturally occurring saccadic eye movements fall within the mid-amplitude range and are made without head movements.

The above description by Lebedev et al. (19) of the main sequence suggests a model resembling an S curve with peak velocity displaying a fairly flat slope over very small and very large amplitudes. Meanwhile, the definition of saccadic mean velocity in relation to their duration and amplitude given in (6) suggests that such a model conform to the observed near-linearity of the duration-amplitude relation e.g., as given by the linear expression for MT in (1a). Finally, parameters of the model should be easy to interpret. A model derived from the logistic function satisfies all of these requirements.

**Figure 2:**
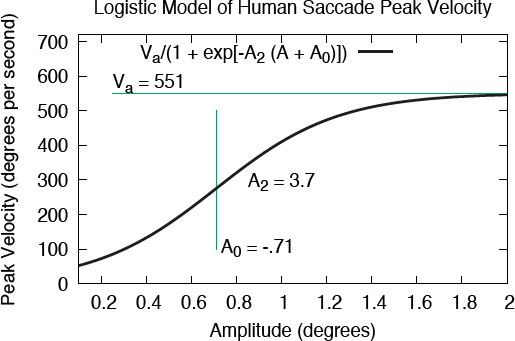
Interpretation of logistic function parameters

A logistic function model for saccadic velocity can be expressed as


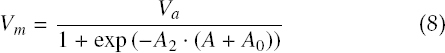


where A*2* is the sigmoid curve’s steepness (slope), A*0* is the sigmoid’s midpoint, Va is the curve’s asymptotic maximum (see Figure 2). Unfortunately, while the logistic function can be made to fit peak velocity data, it does not satisfy the remaining requirement of near-linearity when using it to express the duration- amplitude relation. The problem rests in the resultant expression for MT being fixed at 0 for the y-intercept. To achieve the desired flexibility in the model’s expressivity, the logistic function is augmented with what resembles an inverse-linear component


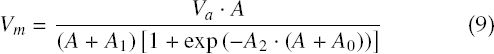


which we term the inverse-linear logistic model of saccadic peak velocity (save for the constant scalar K as per (7)). The additional parameter A*1* produces a shift of the function, which is made clear in the function’s expression for the duration-amplitude relation.

Using (6) and solving for MT=A/V_m_ yields


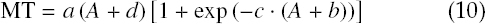


with a = 1/V_a_, b = k_0_ - A_0_, c = k_2_ · A_2_, and d = k_1_ · A_1_. Choosing parameters Va = 551, A*0* = -0.71, A! = 4.0, A*2* = 3.70, k*0* = 14.99, k*1* = 3, k*2* = 0.48, and K = 2.5 · Va as per (7) to produce MT = K · Vğ, yields the functions plotted in Figures1(a) and 1(b) that visually match the functions fit by Baloh et al. (3) with V_a_ = 551 and A_0_ = 14 (see above) in the large-amplitude range.

**Figure 3:**
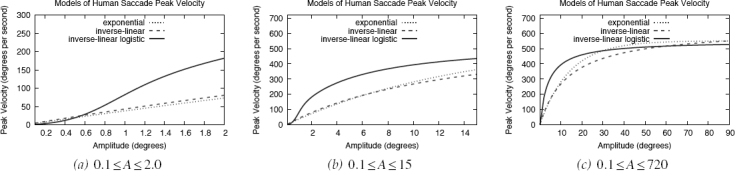
Inverse-linear logistic model of the saccadic main sequence: (a) small-amplitude range; (b) mid-amplitude range; (c) large-amplitude range. Note that these plots are of the same three main sequence models, shown at three different amplitude ranges. The peak velocity range of the small-amplitude plot (a) is reduced to make the inflection point visible (at about 0.7^°^ amplitude) which neither of the exponential nor inverse-linear functions can adequately represent.

The goodness of fit problem of the exponential and inverse-linear functions may lie in the small-amplitude (e.g., microsaccadic) range. Examining this range, it is clear that these two functions and the inverse-linear logistic functions diverge, see Figure 3. In this instance it may appear that the inverse-linear logistic function reaches asymptote too quickly. Because none of the fits were based on actual microsaccade data, it is difficult to tell which curves are growing at an appropriate rate. Inspecting Figure 3(a) and focusing on the exponential and inverse-linear fits would suggest that microsaccades reach peak velocities of less than 100° per second. This is not very likely. Reaching asymptote at about 150° per second is probably more realistic. More over, neither of the exponential nor the inverse-linear functions possess an initial inflection seen at very low amplitudes (the bottom part of the S curve).

**Figure 4:**
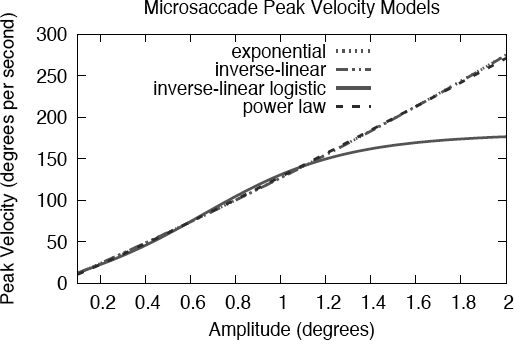
Functions fit to microsaccade data of Experiment 2 (see text). Note that only the inverse-linear logistic function is able to fit both inflections at lower and higher amplitudes (e.g., ~0.4° and ~1.2°). Data to which these fits were made are shown in Figure 8(a), see text.

Do microsaccades exhibit an inflection at very low amplitudes? Examining fits to microsaccade data (see Figure 4) suggests that there is an inflection point at very low amplitudes (about about 0.5^°^ amplitude). At about 1.5° a microsaccadic asymptote is apparent. The exponential and inverse-linear models, when fit to microsaccadic peak velocities, “miss the turn” at both locations. The S inherent in the logistic function affords a better fit. Below we compare statistical fits to microsaccade data from three experiments conducted that were in part designed to capture microsaccades.

## Empirical Methodology

To compare and contrast saccadic main sequence model fits, we examine data captured from three eye tracking experiments.The first two were designed to replicate the experiment of Siegenthaler et al. (29) but using eye trackers sampling at two different rates (500 Hz and 300 Hz). The study was originally designed to test microsaccadic response to task difficulty and mental fatigue, necessitating exclusion of saccades (controlled within the experimental procedure; see below). The third experiment considers microsaccades and saccades from an experiment conducted to evaluate affective response to images of faces gradually changing shape (morphing) to express one of several emotions. Below we provide detailed description of the study methodologies, including a brief review of experimental design with independent and dependent measures, procedure, participants, and equipment. Our focus here is not so much on the analyses of results pertaining to the study hypotheses, rather we are concentrating on characteristics of saccades and microsaccades observed in each of the three experiments.

### Experiments 1 and 2: Microsaccades

The first two experiments closely followed the experimental design of Siegenthaler et al. (29). In each of our two experiments we used a 3 χ 6 within-subjects design where the first fixed factor was task type (Difficult vs. Easy vs. Control) and the second fixed factor was Time-on-Task where six blocks of trials within the experimental procedure constituted the six levels of this fixed factor. In the Difficult and Easy tasks, participants were asked to perform difficult and easy mental calculations, while in the Control task, they were not asked to perform any mental calculations at all (see Experimental Procedure below).

Following Siegenthaler et al. (29), we focused on microsaccade magnitude and rate, and following Di Stasi et al. (9), we analyzed fits of the relationship between microsaccadic amplitude and peak velocity.

*Experimental Procedure* Following signing of a consent form and completion of an online demographic questionnaire, participants sat at the eye-tracking computer with their head stabilized by a chin rest. After making sure participants were comfortable, a 5-point eye tracker calibration was performed. Experimental tasks started when the average calibration error was lower than 0.5° visual angle.

The experimental procedure followed that of Siegenthaler et al. (29), described here for completeness. Three types of number counting trials, Difficult, Easy, and Control, were grouped into 6 blocks, giving 18 trials total. Each block started with the Control trial, followed by the Easy and Difficult trials in counterbalanced order. Between each block, participants were asked to take a short break lasting 2-5 minutes; they were not allowed to start the next block until at least 2 minutes had elapsed.

Each trial started with an instruction screen and included a break at the end of each of the six blocks. In the Difficult trials, participants were asked to mentally count backwards, as fast and accurately as possible, in steps of 17 starting at one of the following 4-digit numbers drawn randomly from this set: {1375, 8489, 5901, 5321,4819,1817}.

The Easy and Control trials were constructed similarly to Difficult trials, but differed in task performance and initial instructions. In the Easy tasks, participants were instructed to mentally count forward, as fast and accurately as possible, in steps of 2 starting at one of the following 3-digit numbers drawn randomly from this set: {363, 385,143, 657, 935,141}. In the Control trials, participants were asked just to gaze at the fixation point with no mental task assigned.

When doing the experimental task, participants were asked to gaze at the fixation point appearing at screen center. Whenever their gaze shifted 3° visual angle away from the fixation point a warning beep sounded.

During each trial, participants were prompted four times to enter in their current number in a text box shown on the screen. A limit of 9 seconds was given for providing the entry. Three prompts appeared at random times during each trial, and the fourth at the very end of the trial. The gap between prompts was a minimum of 15 seconds and a maximum of 80 seconds.

Since we do not focus specifically on the impact of mental calculations on the microsaccade-peak velocity relationship, only data from the Control tasks were selected for analysis.

*Participants. * Participants (N = 17) volunteered for Experiment 1, recruited verbally and via social media. Due to problems with eye tracker calibration or misunderstanding of the task by participants (i.e., in at least one case the participant stopped counting after one iteration, see Experimental Procedure above), data from four subjects were discarded resulting in a final sample of N = 13. Data from 7 males and 6 females aged between 20 and 40 years old (M = 29.77; SD = 7.15) was used in the analysis. All participants reported normal, uncorrected vision.

Participants (N = 10) volunteered for Experiment 2, recruited verbally. All participants reported normal, corrected or uncorrected vision.

**Figure 5:**
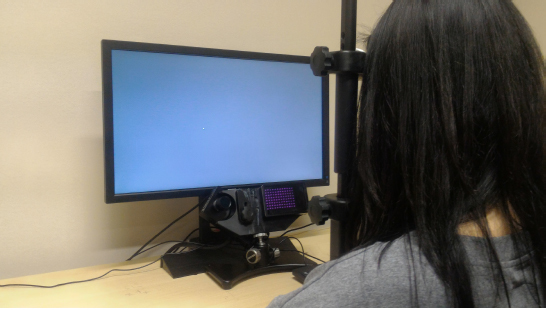
. Experimental setting featuring the SR Research EyeLink 1000 eye tracker and chin rest.

*Experimental Setting and Apparatus. * In Experiment 1, an SR Research EyeLink 1000 eye tracker was used for eye tracking data acquisition. Eye movements were recorded binocularly at a sampling rate of 500 Hz. Each participant’s head was stabilized with a chin rest during the entire experimental procedure, see Figure 5. The accuracy of the EyeLink 1000 tracker is reported by the manufacturer as 0.25°-0.5° visual angle on average, with microsaccade resolution of 0.05°.

The experimental procedure was controlled by a personal computer connected to the eye-tracking computer. Visual stimuli were displayed on a 24 inch computer screen with 1920x1080 resolution at a viewing distance of 57 cm. Responses made by participants were performed on a standard numerical keyboard connected to the stimuli presentation computer and placed by the participant’s dominant hand.

**Figure 6:**
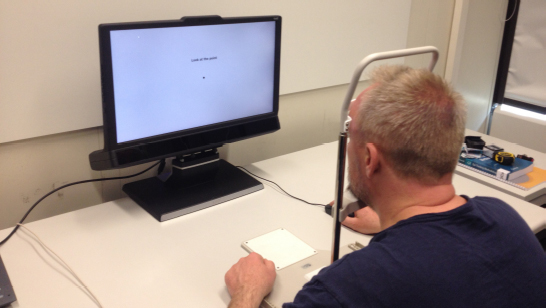
Experimental setting featuring the Tobii TX300 eye tracker and chin rest.

In Experiment 2, conditions were similar, except that a 300 Hz eye tracker from Tobii was used. As in Experiment 1, each participant’s head was stabilized with a chin rest during the entire experimental procedure, see Figure 6. The accuracy of the Tobii TX300 tracker is reported by the manufacturer as 0.3°-0.6° visual angle on average.

### Experiment 3: Microsaccades & Saccades

Experiment 3 differed from the first two considerably. Its objective was to study facial affect recognition and followed procedures similar to those of Schönenberg, Mayer, Christian, Louis, and Jusyte (28). For the purposes of this analysis, what is most important is that participants viewed a computer monitor and maintained their gaze in a fairly central location. Unlike the first two experiments, Experiment 3 did not restrict eye movement and allowed freedom to visually inspect the stimulus. Recall that Experiments 1 and 2 restricted gaze to a central point on the screen location with an audible reprimand sounding whenever gaze strayed too far away (3°) from center.

Although there are considerable differences in tasks and experimental objectives, here we are mainly concerned with finding suitable functions to fit microsaccadic and saccadic peak velocity profiles.

*Experimental Procedure. * The experimental procedure consisted of participants watching a parametrically varied image sequence (morphed animation) of a human face. The animated morph task depicted a video sequence of a neutral face slowly developing into one of six basic emotions (fear, sadness, anger, happiness, disgust, surprise). Faces were presented in the center of the computer screen and subtended 16.8° visual angle in width and 21.1° visual angle in height.

Participants were instructed to press a button as soon as they were able to identify the emerging emotion. The sequence was then immediately stopped, the face disappeared, and participants were presented with a mask instructing them to indicate which emotion they had identified by selecting one of the six verbal categories via a button press. The morph intensity level at the time of the button press, as well as the participant’s judgment of the emotional expression, was recorded.

For the purposes of the analysis of eye movements in the present article, we ignore dependent variables pertaining to affect recognition, e.g., the intensity of emotional expression at the time of the button press, correctness of response, etc.

*Participants. * Data were collected from three groups of participants: two clinical groups and a Control group of people who reported no mental disorders (N = 20). Note that for the sake of internal coherence of the present analysis, we focus only on data from the Control group since we are not interested in the differences between research samples.

*Experimental Setting and Apparatus. * In Experiment 3, the same type of eye tracker was used as in Experiment 1, model EyeLink 1000 from SR Research. Eye movements were recorded binocularly at a sampling rate of 500 Hz.

Visual stimuli were displayed on a 19 inch computer screen with 1024x768 resolution at a viewing distance of 60 cm.

## Event Detection

Both saccade and microsaccade detection algorithms were based on the work of Engbert and colleagues (Engbert et al., 13; Engbert, 11; Engbert & Kliegl, 12). Note that the algorithms differ, although both were ostensibly originally designed to work on raw gaze data (raw meaning insofar as not being subject to any type of event detection by the eye tracker, hence consisting solely of (*x(t), y(t))* data). We use Engbert et al.’s (13) algorithm in Experiment 3 to detect saccades, but our version of microsaccade detection differs from that of Engbert (11) in that we apply his algorithm only to segments of raw gaze points identified as fixations, via velocity-based (I-VT) event detection (Salvucci & Goldberg, 26) by the Savitzky and Golay (27) filter following Nyström and Holmqvist (23). The motivation for doing so is for eventual real time applications. Because fixation event detection can be applied to streaming data, subsequent filtering for microsaccade detection should incur only small costs in additional processing and latency, thus, we conjecture, making real-time microsaccade detection feasible. Alternatively, e.g., following Otero-Millan, Macknik, Langston, and Martinez-Conde (25). and using only one algorithm to describe the whole range of events as saccades or microsaccades varying along a continuum as a function of the size of the scene being scanned, typically requires the entire raw gaze data set, precluding real-time implementation.

### Microsaccade Detection

Microsaccades can be detected when gaze is fixed on a stationary object, i.e., during a fixation. Given a sequence of raw gaze points identified within a fixation, we adapt a version of Engbert and Kliegl’s (12) algorithm for the detection of microsaccades.

The algorithm proceeds in three steps. First, the gaze position time series is transformed to velocities via


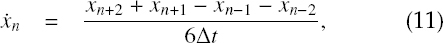


but is done separably for *x(t)* and *y(t)*. Equation (11) represents a moving average of velocities over 5 sample range (skipping the center point at n, giving a n + 2 - n + 2 + 1 = 5 sample range). As Engbert and Kliegl note, due to the random orientations of the velocity vectors during fixation, the resulting mean value is effectively zero. Microsaccades, being ballistic movements creating small linear sequences embedded in the rather erratic fixation trajectory induced by small drifts, can therefore be identified by their velocities, which are clearly separated from the kernel of the distribution as “outliers” in velocity space. We took 6Δt to mean the sampling period Δt multiplied by constant scalar 6. This would be reasonable given the assumption of a uniform sampling rate (i.e., Δt = 1 /*f* where *f* is the sampling frequency in Hertz). In practice, because actual sampling periods are not uniform (e.g., due to network or multitasking architecture issues), we do not use the 6Δί in the denominator, rather we take the difference in timestamps of the actual data, i.e., we replace 6Δt by *^(t^n*+2 *^- t^n*-2^)^.

Second, computation of velocity thresholds is based on the median of the velocity time series to protect the analysis from noise. A multiple of the standard deviation of the velocity distribution is used as the detection threshold (Engbert, 11),


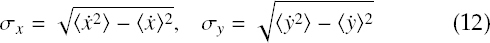


where <·> denotes the median estimator. Detection thresholds are computed independently for horizontal and vertical n_y_ components and separately for each trial, relative to the noise level, i.e.,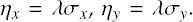 Like Engbert and Kliegl (12), we used λ = 6 for microsaccade detection in Experiments 1 and 3 and then λ = 3.6 in Experiment 2 (to reflect the drop in sampling rate from 500 Hz to 300 Hz)^3^ and we assume a minimal microsaccade duration of 6 ms (three data samples at 500 Hz) in Experiments 1 and 3 and 6.6 ms (three data samples at 300 Hz) in Experiment 2.

Following Engbert (11) as a necessary condition for a microsaccade, we require X and y fulfill the criterion 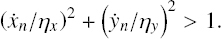


Microsaccade amplitude is defined as mean displacement amplitude of the sequence of gaze points wherein each gaze point satisfies the above necessary condition for microsaccade labeling. Microsaccade amplitude is reported in degrees visual angle. Given detection of a microsaccade gaze point sequence, the next set of samples comprising a 20 ms intersaccade interval are skipped so as not to count overshoots.

Third, Engbert and Kliegl (12) focus on binocular microsaccades, defined as microsaccades occurring in left and right eyes with a temporal overlap. They exploit binocular information by applying a temporal overlap criterion: if a microsaccade in the right eye starting at time r_1_ is found that ends at time r_2_, and a microsaccade in the left eye begins at time 1_1_ and ends at time 1_2_, then the criterion for temporal overlap is implemented by the conditions r_2_ > 1_1_ and r_1_ < 1_2_. We omit this step as we typically average both left and right gaze points into a single point as would be looked at by a cyclopean eye, i.e., (x(t), y(t)) = ([*x_l_(t)* + *x_r_(t)*]/2, [*y_l_(t)* + *y_r_(t)*]/2).

Engbert and Kliegl (12) assume a stationary eye movement signal, i.e., when fixating an object, e.g., performing a task where gaze is meant to be held steady (e.g., see Siegenthaler et al. (29)). To adapt their algorithm to the general case of a non-stationary eye movement signal, we first detect fixations following Nyström and Holmqvist (23), and use the Savitzky and Golay (27) filter for velocity-based (I- VT (Salvucci & Goldberg, 26)) event detection. The Savitzky-Golay filter fits a polynomial curve of order n via least squares minimization prior to calculation of the curve’s derivative (e.g., 1^st^ derivative (s = 1) for velocity estimation), (Gorry, 16). We used a 3^rd^ degree Savitzky-Golay filter of width 3 with velocity threshold of 100°/s, tuned to the sampling rate of the eye tracker used.

### Saccade Detection

Saccade detection is based on the work of Engbert et al. (13) and proceeds similarly to microsaccade detection with a few minor changes. The algorithm follows the same steps as for microsaccades except that Equation (12) is changed to


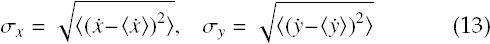


where <·> again denotes the median estimator. The same criterion is used for velocity thresholding, namely 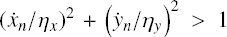 again with 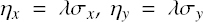, except that for saccades λ is set to 8. For saccade detection Engbert et al. (13) also exploit binocular events, whereas we use the cyclopean averaging as for microsaccades.

Note that to detect saccades, we use an entire raw eye movement time series, and we do not subject the sequence to velocity detection with the Savitzky-Golay filter. Also, we implement a similar criterion for minimum duration saccades as for microsaccades above. In practice we look for a minimum of two successive points (i.e., an edge) to define a saccade. In other words, isolated points in the data stream that are over threshold are not classified as saccades.

## Results

Analysis of results aims to answer two questions:

1. how well does the inverse-linear logistic function (9) fit peak velocity-amplitude data for microsaccades and saccades; and
2. can the inverse-linear logistic function, in its expression for duration (10), adequately describe the linear duration-amplitude relationship of microsaccades and saccades?

Before providing empirical evidence for these queries, in Table 1 we first present descriptive statistics of microsaccades from our three experiments (saccades captured in the third experiment are discussed later). Microsaccadic amplitudes and durations are similar across all three experiments, and are in line with what is found in the literature, e.g., see Otero-Millan, Troncos o, Macknik, Serrano-Pedraza, and Martinez-Conde (24).

**Table 1:** Descriptive statistics of microsaccade parameters: amplitude (deg), peak velocity (deg/sec), duration (sec), and rate (count/sec) from Experiments 1, 2, and 3.

	Mean	SD	Minimum	Maximum	Skewness	Kurtosis
Experiment 1. Fixating @ 500 Hz: microsaccades						

amplitude	0.40	0.16	0.02	1.60	1.21	2.34
peak velocity	70.64	34.97	4.00	215.74	1.19	1.49
duration	0.01	0.01	0.01	0.04	1.51	2.76
rate	4.31	7.2	0.1	55.56	4.13	19.79

Experiment 2. Fixating @ 300 Hz: microsaccades						

amplitude	0.54	0.25	0.06	1.98	0.85	0.63
peak velocity	66.73	35.81	10.38	200.28	0.89	0.22
duration	0.02	0.01	0.01	0.04	1.18	0.78
rate	1.56	3.38	0	33.49	4.92	32.48

Experiment 3. Free-viewing @ 500 Hz: microsaccades						

amplitude	0.48	0.21	0.03	1.55	0.58	0.09
peak velocity	101.01	47.57	5.40	219.63	0.24	-0.79
duration	0.02	0.01	0.01	0.04	0.52	-0.73
rate	3.83	4.34	0.08	55.56	6.45	57.47

However, one should also note in Table 1 a greater microsaccade mean peak velocity in Experiment 3 where participants were allowed to move their eyes over the given stimuli (pictures of faces). This is also consistent with the literature. For example, MartinezConde (21) previously showed that a steady fixation leads to a decrease in microsaccade amplitude (resulting in visual fading).

**Figure 7:**
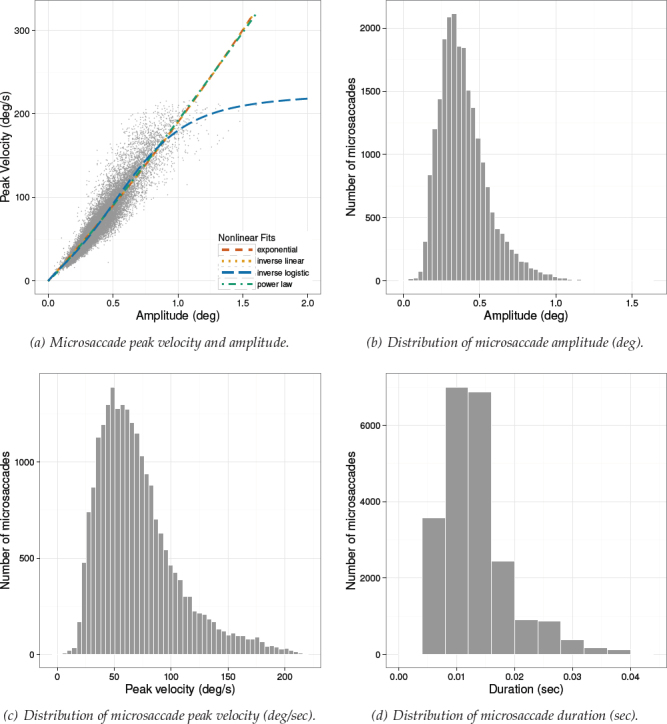
Experiment 1. Distribution of microsaccade amplitude (b), peak velocity (c), and duration (d). Figure (a) depicts the nonlinear relationship between peak velocity and amplitude with different function fits. Data were captured at 500 Hz.

**Figure 8:**
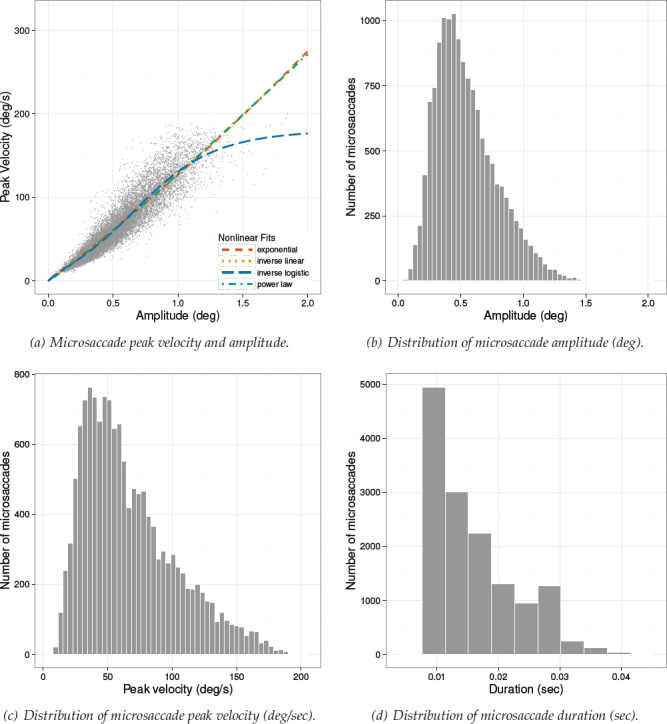
Experiment 2. Distribution of microsaccade amplitude (b), peak velocity (c), and duration (d). Figure (a) depicts the nonlinear relationship between peak velocity and amplitude with different function fits. Data were captured at 300 Hz.

**Figure 9:**
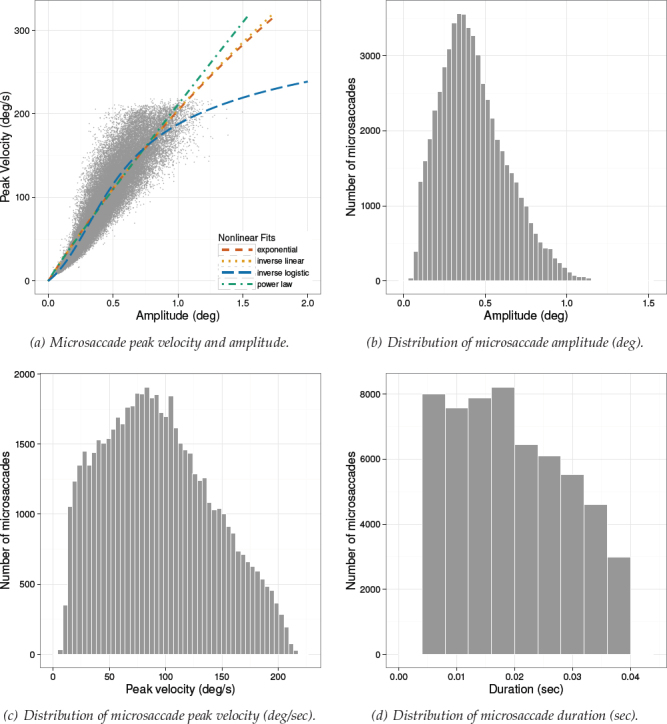
. Experiment 3. Distribution of microsaccade amplitude (b), peak velocity (c), and duration (d). Figure (a) depicts the nonlinear relationship between peak velocity and amplitude with different function fits. Data were captured at 500 Hz.

Microsaccadic distributions and the microsaccadic peak velocity vs. amplitude scatterplots are given in Figures 7-9 for Experiments 1-3, respectively. Plots of the microsaccadic and saccadic peak velocity amplitude relation include different functions fit to the data, for comparison with the inverse-linear logistic function fit. These function fits are discussed below.

### Fitting Peak Velocity-Amplitude

A traditional approach to testing linear model (function) goodness of fit is through linear regression analysis, i.e., estimation of *R*^2^. The computation of *R*^2^, however, relies on estimation of the distance between the observations and the model (residuals), i.e., the line that was determined through least squares minimization (i.e., minimization of the distance), see Boggs, Byrd, and Schnabel (5). For *R*^2^ to be meaningful, the distances computed assume orthogonality between the observations and the line fit to them. For a nonlinear fit, the assumption of orthogonality might not hold (Wolter & Fuller, 33; Stefanski, 30).

Instead of *R*^2^, we examine the relative quality of our nonlinear models through Akaike’s Informationtheoretic Criterion (AIC) (1). What matters is not AIC itself but the difference in AIC between models (ΔAIC). Under this criterion, the model with the smallest AIC exhibits the best fit. Supplementing AIC, we also report results from Vuong’s test, a likelihood- ratio based statistic, for non-nested, nonlinear model comparison (Vuong, 32). Vuong’s also tests for statistical significance between the fits of the models under consideration.

*Microsaccade Peak Velocity-Amplitude. * For microsaccade data from all experiments, the inverse linear logistic function provides the best fit, yielding the smallest AIC. Moreover, as alluded by Lebedev et al. (19), the other nonlinear models show inconsistency in their goodness of fits, see Table 2.

**Table 2:** Tests of nonlinear model fit to microsaccades. For all ΔAIC the inverse-linear logistic model is the base as it yields the smallest AIC criterion. Vuong’s tests (last column) compare all other models to the inverse-linear logistic fit. Data from Experiments 1 (top), 2 (middle), and 3 (bottom).

Model	Residual SE	model df	AIC	ΔAIC	Vuong’s tests
Experiment 1. Fixating @ 500 Hz					

inverse-linear logistic	SEe = 12.13	22347	175008.7	0.0	—
power-law	SEe = 12.52	22349	176419.9	1411.2	z = 7.59, p < 0.001
exponential	SE_e_ = 12.66	22349	176893.8	1885.1	z = 9.85, p < 0.001
inverse-linear	SE_e_ = 12.66	22349	176913.2	1904.6	z = 10.05, p < 0.001

Experiment 2. Fixating @ 300 Hz					

inverse-linear logistic	SEe = 13.29	14146	113376.6	0.0	—
power-law	SEe = 13.68	14148	114187.7	811.1	z = 7.74, p < 0.001
exponential	SEe = 13.79	14148	114412.3	1035.7	z = 9.57, p < 0.001
inverse-linear	SEe = 13.79	14148	114421.3	1044.7	z = 9.72, p < 0.001

Experiment 3. Free viewing @ 500 Hz					

inverse-linear logistic	SEe = 19.41	57027	500146.2	0.0	—
exponential	SE_e_ = 20.32	57029	505383.5	5237.3	z = 41.32, p < 0.001
inverse-linear	SEe = 20.34	57029	505467.8	5321.6	z = 41.33, p < 0.001
power-law	SEe = 20.56	57029	506687.3	6541.1	z = 41.33, p < 0.001

We should point out that for the prolonged fixation of Experiments 1 and 2, the rank ordering of the AIC is the same. In these experiments, the inverse-linear model gave the worst fit. In Experiment 3, where the eyes were free to move, the power-law function gives the worst fit. Similar differences in microsaccades between fixating and free-viewing were noticed by Otero- Millan et al. (24), although they did not fit different functions to the data

Pairwise comparison of the inverse-linear logistic function with all others with Vuong’s test (with AIC correction) revealed a statistically significant difference in fits, with the inverse-linear logistic function giving better fits in all cases, see Table 2. Table 3 gives the parameter estimates for the inverse-linear logistic function in each of the three experiments. Significance tests indicate that all parameters differed significantly from zero.

**Table 3:** Non-linear regression coefficients and their significance tests for the inverse-linear logistic function fit to ***microsaccades.***

Parameter	Estimate	SE	t-test
Experiment 1. Fixating @ 500 Hz			

V_a_	236.22	4.71	t(22347) = 50.18, p < 0.001
A_1_	0.16	0.02	t(22347) = 8.25, p < 0.001
A_2_	3.93	0.07	t(22347) = 53.76, p < 0.001
A_0_	-0.48	0.007	t(22347) = -71.73, p < 0.001

Experiment 2. Fixating @ 300 Hz			

V_a_	188.58	3.87	t(14146) = 48.62, p < 0.001
A_1_	0.11	0.02	t(14146) = 5.33, p < 0.001
A_2_	3.37	0.07	t(14146) = 47.28, p <0.001
A_0_	-0.64	0.008	t(14146) = 81.63, p < 0.001

Experiment 3. Free viewing @ 500 Hz			

V_a_	323.29	8.95	t(57351) = 36.11, p < 0.001
A_1_	0.71	0.05	t(57351) = 15.39, p < 0.001
A_2_	5.79	0.12	t(57351) = 47.47, p < 0.001
A_0_	-0.18	0.008	t(57351) = 22.96, p < 0.001

**Figure 10:**
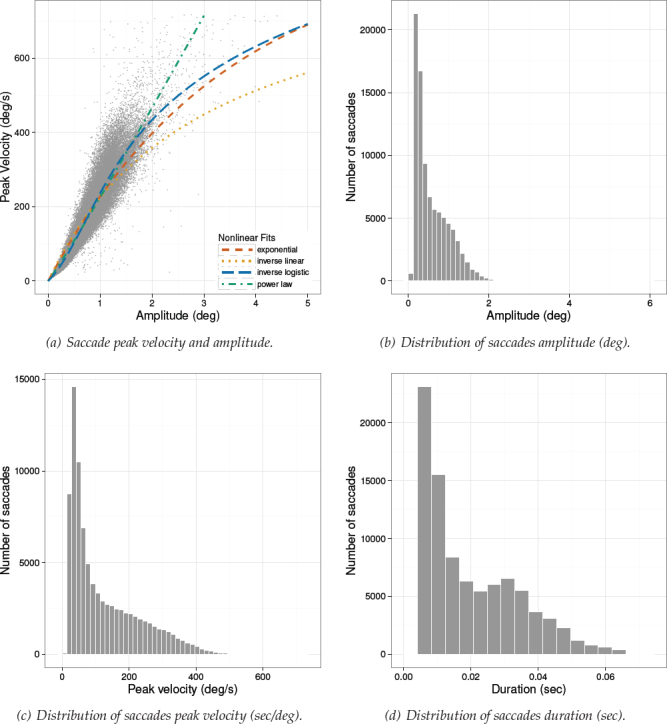
Experiment 3. Distribution of saccade amplitude (b), peak velocity (c), and duration (d). Figure (a) depicts the nonlinear relationship between peak velocity and amplitude with different function fits. Data were captured at 500 Hz.

*Saccade Peak Velocity-Amplitude. * Saccades were captured only in Experiment 3 because only in this reported study participants could freely move their eyes. There were N *=* 89020 saccades detected in total. Detailed descriptive statistics for saccades are given in Table 4. Notice that the maximum amplitude observed was over 6 degrees visual angle, placing these saccades in the small- to mid-amplitude range. Saccade distributions and the saccade peak velocity-amplitude scatter- plot from Experiment 3 is given in Figure 10, along with depictions of functions fit to the data in Figure 10(a).

**Table 4:** Descriptive statistics of saccade parameters: amplitude (deg), peak velocity (deg/sec) and duration (sec) from Experiment 3. There were N = 89020 in total saccades detected.

	Mean	SD	Minimum	Maximum	Skewness	Kurtosis
	Experiment 3. Free viewing @ 500 Hz: saccades						

amplitude	0.74	0.54	0.07	6.31	1.17	1.18
peak velocity	160.19	135.47	10.35	719.31	1.16	0.55
duration	0.02	0.01	0.01	0.07	0.89	-0.07

The same nonlinear goodness of fit analysis was carried out for the saccadic peak velocity-amplitude relation as for microsaccades, above. Similar to microsaccades, the AIC shows that the function providing the best fit is the inverse-linear logistic function, see Table 5. The inverse-linear function gives the worst fit Voung’s tests, also in Table 5, suggest that the inverse linear logistic function fits the data significantly better than any of the other functions. Table 6 lists the parameter estimates for the inverse-linear logistic function.

**Table 5:** Tests of nonlinear model fits to saccades. For all ΔAIC the inverse-linear logistic model is the base as it yields the smallest AIC criterion. Vuong’s tests (last column) compare all other models to the inverse-linear logistic fit. Data from Experiment 3 (free viewing @ 500 Hz).

Model	Residual SE	model df	AIC	ΔAIC	Vuong’s tests
inverse-linear logistic	SE_e_ = 37.59	89016	898344.4	0.0	—
power-law	SE_e_ = 40.20	89018	910293.4	11949.0	z = 14.39, p < 0.001
exponential	SE_e_ = 45.68	89018	933030.7	34686.3	z = 80.83, p < 0.001
inverse-linear	SE_e_ = 53.34	89018	960638.5	62294.1	z = 99.72, p < 0.001

**Table 6:** Non-linear regression coefficients and their significance tests for the inverse-linear logistic function fit to ***saccades.***

Parameter	Estimate	SE	t-test
Va	895.06	18.24	t(89016)= 49.07, p < 0.001
A1	1.56	0.08	t(89016)= 18.03, p < 0.001
A2	1.70	0.01	t(89016)=156.46, p < 0.001
A0	-0.63	0.02	t(89016)=-26.95, p < 0.001

*Superpositioning Microsaccades and Saccades. *To test whether the inverse-linear logistic function provides a good fit across microsaccade and saccade amplitude ranges, we superpositioned both types of eye movements into a single data set and followed the same analytical procedure as above for the separate microsaccade and saccade analysis.

Results of AIC analysis suggests the inverse-linear logistic function goodness of fit is maintained across the small and mid-amplitude ranges, see Table 7. Table 7 also shows results of Voung’s tests which again indicate a statistically significant better fit of the inverse- linear logistic function compared to the others. Table 8 lists the parameter estimates for the inverse-linear logistic function.

**Table 7:** Tests of nonlinear model fits to ***saccades and microsaccades.*** For all ΔAIC the inverse-linear logistic model is the base as it yields the smallest AIC criterion. Vuong’s tests (last column) compare all other models to the inverse-linear logistic fit. Data from Experiment 3 (free viewing @ 500 Hz).

Model	Residual SE	model df	AIC	ΔAIC	Vuong’s tests
inverse-linear logistic	SE_e_ = 32.69	148565	1457793.0	0.0	—
power-law	SE_e_ = 34.05	148567	1469847.0	12054.0	z = 10.808, p < 0.001
exponential	SE_e_ = 39.10	148567	1511004.5	53211.5	z = 82.466, p < 0.001
inverse-linear	SE_e_ = 45.48	148567	1555887.6	98094.6	z = 108.325, p < 0.001

**Table 8:** Non-linear regression coefficients and their significance tests for the inverse-linear logistic function fit to superpositioned ***microsaccades and saccades.***

Parameter	Estimate	SE	t-test
Va	793.48	8.73	t(148565)= 90.86, p < 0.001
A1	0.91	0.04	t(148565)= 25.34, p < 0.001
A2	1.49	0.01	t(148565)=133.21, p < 0.001
A0	-0.88	0.01	t(148565)=-64.64, p < 0.001

### Fitting the Duration-Amplitude Main Sequence

While empirical evidence thus far shows that the inverse-linear logistic function produces the best fit to the relation between peak velocity and amplitude, it may not be immediately obvious whether the function can also serve to express the classic linear relation between duration and amplitude, i.e., the main sequence (Bahill et al., 2).

We have shown mathematically that the inverse linear logistic expression (10; restated below) can be used to describe the duration-amplitude relation. Here, we test this assertion empirically by comparing the expression’s fit to that of the linear function given by (1a). To do so, we use the data from the above three experiments, this time plotting duration vs. amplitude and compare the linear fit of (1a)


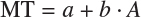


to that of (10).

For the analysis, the inverse-linear logistic function is manually fit to the data, while the linear fit is obtained automatically through linear model fitting in software (R). Linear curve fitting for the inverse-linear logistic function proceeds by first automatically fitting expression (9) for peak velocity-amplitude. This was performed automatically in software (using R’s nls() function, see Fox and Weisberg (14). Recall expression (9)


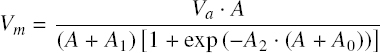


is converted to expression (10)


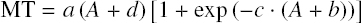


by using (6) and solving for MT = *A/V_m_* with 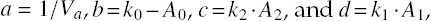, using constant e such that $$$ as per (7) to produce MT = K V_m_ Coefficients k_Q_-k*2* and K, given in Table 9, were obtained by manually fitting the inverse-linear logistic function to each of the data sets used for fitting the peak velocity amplitude relation, but replotted using duration vs. amplitude. In each instance, a linear fit of (1a) was obtained automatically via minimization of least squares (in R) for comparison to (10).

**Table 9:** Linear coefficients for the inverse-linear logistic function fit to duration-amplitude of all data sets.

Experiment	Parameter			
	k0	k1	k2	K
Exp. 1. Fixating @ 500 Hz (microsaccades)	.516223	3.51575	2.29179	0.0132· V_a_
Exp. 2. Fixating @ 300 Hz (microsaccades)	.336892	14.32469	2.866794	0.0076· V_a_
Exp. 3. Free viewing @ 500 Hz (microsaccades)	.769782	0.995477	1 . 70962	0.01685· V_a_
Exp. 3. Free viewing @ 500 Hz (saccades)	.369424	0.262244	5.278945	0.018 ·V_a_
Exp. 3. Free viewing @ 500 Hz (superpositioned microsaccades and saccades)	.369424	0.262244	5.278945	0.0177· V_a_

In line with expectations, for microsaccadic data in Experiments 1, 2, and 3 the linear and inverse-linear logistic functions fit the data similarly. Comparison of the AIC for linear (AIC = -170475.2) and inverse-linear logistic (AIC = -170475.2) functions for microsaccades in Experiment 1 showed that both models fit similarly to the data. Analyses of Experiment 2 also showed similar fits for both linear (AIC = -103460.1) and the inverselinear logistic model (AIC = -103460.1). Analyses of Experiment 3 also yielded similar results for the linear (AIC = -388684.6) and inverse-linear logistic models (AIC = -388684.7).

Similar AIC results were found for saccades fit by the linear (AIC = -576451.0) and inverse-linear logistic (AIC = -576450.7) models in Experiment 3. Combined saccade and microsaccade data also yielded similar fits by the linear (AIC = -956121.2) and inverse-linear logistic (AIC = -956120.7) models.

AIC statistics for each of the pairs of the linear and inverse-linear logistic fits are nearly identical, suggesting that both models are indistinguishable. One may argue, as we do above, that AIC statistics are more suitable to nonlinear goodness of fit estimates and that in this instance of testing linear models, the traditional approach to testing goodness of fit is through linear regression analysis, i.e., estimation of *R*^2^. Linear regression statistics of each pair of models is provided in Table 10, which shows identical *R*^2^ for the model pairs in each of the five data sets.

**Table 10:** Linear fit analysis for the inverse-linear logistic and linear model fit to all data sets.

Linear Model		Inverse-Linear Logistic Model	
F statistic and its p value	Model R^2^	F statistic and its p value	Model R^2^
Experiment 1. Fixating @ 500 Hz: microsaccades			

F (1,22349) = 3723, p < 0.01	R^2^ = 0.1431	F(1,22349) = 3723, p < 0.01	R^2^ = 0.1431

Experiment 2. Fixating @ 300 Hz: microsaccades			

F (1,14148) = 1254, p < 0.01	R^2^ = 0.0814	F(1,14148) = 1254, p < 0.01	R^2 ^= 0.0814

Experiment 3. Free Viewing @ 500 Hz: microsaccades			

F (1,57029) = 10760, p < 0.01	R^2^ = 0.1588	F(1,57029) = 10760, p < 0.01	R^2 ^= 0.1588

Experiment 3. Free Viewing @ 500 Hz: saccades			

F (1,89018) = 92440, p < 0.01	R^2^ = 0.5094	F (1,89018) = 92440, p < 0.01	R^2 ^= 0.5094

Experiment 3. Free Viewing @ 500 Hz: microsaccades and saccades			

F(1,148567) = 99620, p < 0.01	R^2^ = 0.4014	F(1,148567) = 99620, p < 0.01	R^2 ^= 0.4014

In sum, when applied to the amplitude-duration relation, the inverse-linear logistic function fits the data as well as the classically assumed linear expression. The inverse-linear logistic function fit extends the main sequence from the microsaccadic small to the saccadic mid-amplitude range.

## Discussion

Our results indicate that the inverse-linear logistic function provides the best fit to the peak velocity amplitude relation in both small- and mid-amplitude ranges. We should note that due to the nature of the experiment conducted allowing free eye movement, the range of eye movements was fairly limited, extending to about 6° instead of 15° demarked by Lebedev et al. (19). Nevertheless, we are confident that the inverse-linear logistic function will serve as a good model of peak velocity-amplitude across all ranges of saccadic amplitudes. Our goodness of fit analysis over the combination of observed microsaccades and saccades shows that the function gives a good fit when the two data sets are superpositioned. Mathematically, the logistic function is linear in its midsection, hence in terms of fit, it is no worse than the other alternative nonlinear functions. What is of primary concern is the flattening of the curve at the low and high ranges (i.e., the asymptotes). Neither of the other nonlinear models have this flexibility, which makes the logistic function more suitable.

The inverse-linear logistic function, while requiring additional parameters over other models, offers greater flexibility in its fit to peak velocity across a wider range of amplitudes. This is due to its logistic component which allows greater control of the function particularly at the low- and large-amplitude ranges where peak velocity tends to asymptote. In the mid- amplitude range, the logistic component of the function allows control of the functions adherence to the observed slope of peak velocity.

The inverse-linear logistic function can also be used to express the expected linearity of the duration- amplitude relation. Our results show that the linear fit afforded by the function is no worse than, and in the least-squares sense identical to, the established linear main sequence.

## Limitations

One limitation of our approach involves our “cyclopean simplification” wherein we average the left and right gaze points into a single point prior to microsaccade detection. This averaging may distort the shape of the main sequence. However, our examination of the inverse-linear logistic function was made with the assumption of the suitability of the exponential, power- law, and inverse-linear fits, as has been demonstrated in the past. Because these latter models also fit the main sequence produced by our algorithm, it is likely that whatever distortion may have been introduced, if any, did not unfairly disadvantage the other functions. Still, our choice of algorithmic simplification should be examined with greater scrutiny in future evaluation of the inverse-linear logistic function.

## Conclusion

We have derived a new model for the main sequence of eye movements, based on an inverse-linear logistic function. We have shown the model’s robust performance when applied to three distinct eye movement data sets, captured at three different laboratories using different eye trackers running at either 300 or 500 Hz. We have shown that the inverse-linear logistic function suitably fits both peak velocity-amplitude and duration-amplitude relations over the low- to mid- amplitude range. We are confident the function will suitably extend over a wide range of eye movement amplitudes. A particularly usable aspect of the model is its demonstrated capacity to simultaneously fit microsaccades and saccades when superpositioned together.

## Footnotes

1 Other variations of the linear main sequence include a = 23, b = 2.7 from Collewijn et al. (1988) and a e [20,30] ms., b e [2, 2.7] ms/deg from Lee, Badler, and Badler (20) and Gu, Lee, Badler, and Badler (17) in their graphical simulation of saccades known as *Eyes Alive*.

2 Carpenter (7) states, “The situation is rather like that of a man falling off a cliff: at first, acceleration dominates, and his peak velocity depends on how far he falls. But if his drop is a long one, most of the way he will befalling at his terminal velocity, so the duration of his fall will be in proportion to the height of the cliff.”

3 Mergenthaler (22) discusses how the choice of λ sub- stantially affects the number of detected microsaccades. As λ increases, the number of detected microsaccades decreases.
